# Individual versus team heart rate variability responsiveness analyses in a national soccer team during training camps

**DOI:** 10.1038/s41598-020-68698-5

**Published:** 2020-07-16

**Authors:** Alejandro Muñoz-López, José Naranjo-Orellana

**Affiliations:** 1 Departamento de Motricidad Humana y Rendimiento Deportivo, Education Sciences School, University of Seville, Desk 4.78, c/Pirotecnica s/n, 41013 Seville, Spain; 2 Sports and Computers Department, University Pablo de Olavide, Seville, Spain

**Keywords:** Health care, Medical research

## Abstract

Heart rate variability (HRV) analyses can be performed using group or individual changes. Individual changes could be of potential interest during training camps for national soccer teams. The purpose of this study was to compare whether analysis of individual daily HRV could detect changes in cardiac autonomic responses during training camps for national soccer teams. During two different training camps, 34 professional soccer players were monitored daily over 9 days, using heart rate monitors. Players were divided into First Eleven (those who participated in the main squad) or Reserves. Daily HRV was individually analyzed using a day-to-day method or a baseline (days prior to first match) method, using the smallest worthwhile change and the typical error in the estimate to establish a trivial (random change) zone. Group changes were also analyzed using an ANOVA one-way repeated measures test. Players’ responsiveness was classified as High-, Low- or Non-response depending on individual changes. Both analyses showed substantial daily individual changes after playing a soccer match, regardless of the group. However, group changes showed that only First Eleven players had significant changes after playing a soccer match. In conclusion, individual daily HRV analyses are useful in detecting individual changes in professional soccer players.

## Introduction

Cardiac autonomic response (CAR) can be used to monitor the cardiovascular adjustments made after exercise^1–3^, which are mediated by the autonomic nervous system^4^. Professional soccer players have shown impairments in physiological and performance measures after playing soccer matches^3,5,6^, especially during congested fixture periods^7,8^, which are typical during training camps for national soccer teams^9^. Therefore, monitoring post-match recovery requires tools that are sensitive to match-induced fatigue to help practitioners make decisions on a daily basis ^5^. However, not only do the tools or variables themselves have an impact in the analysis; so does the method of analysis employed^10^.

Using a simple heart rate monitor (HRM)^11^, heart rate variability (HRV) can be monitored to assess CAR during training cycles in soccer players^12–14^. CAR can be studied by assessing parasympathetic and sympathetic vagal activity via time-domain variables analysis^15^. LnRMSSD is a time-domain variable that reflects parasympathetic activity^2^, and more recently, the Stress Score (SS), has been proposed to reflect sympathetic activity in soccer players^16^. An increase in parasympathetic activity usually reflects a positive adaptation to training^2,17^, which can typically occur after a taper period as sign of functional training adaptation and part of the super-compensation cycle^11^. We have previously shown that parasympathetic activity (as measured via the natural logarithm of the root mean squared standard deviation ((LnRMSSD) did not change during typical training days at a training camp for a professional national soccer team. However, it significantly increased 48 h after playing a soccer match, but only in players who played in the main squad (First Eleven)^9^.

To assess possible changes in human performance and/or fatigue after exercise, a variety of analytical methods have been used^1,3,17–19^. Some authors have highlighted the utility of analyzing individual responsiveness after exercise^18–20^. As Hopkins noted^10^, one of the most important aspects of experimental research is that a treatment aimed at changing subjects’ physiology (e.g. playing a soccer match^21^) may have outcomes that are beneficial, harmful, or ineffective in different individuals. However, traditionally, HRV changes, even on a daily basis, have been performed on the basis of group changes in soccer players^6,22–24^, which are ineffective in detecting individual players’ responses. In addition, individual responses can include athletes who show exceptionally large responses (high responders) and individuals who show exceptionally small responses (low responders)^20^. Some authors have now started to use individual analysis methods in order to track individual changes^1,11,25^.

Interest in more practical measures of fatigue monitoring has been growing, with the aim of helping practitioners to solve problems arising from real-world scenarios^21^. When athletes are evaluated at team level, fatigued athletes may be overlooked^26^. In addition, it has been shown that changes in aerobic fitness are likely different when group analyses are perform in contrast to individual analyses^27^ Therefore, it is suggested that HRV be monitored on an individual basis^28^, to discern how athletes are tolerating match stress at a personal level^1^. Monitoring individual responses can provide coaches with a practical implementation of daily HRV monitoring to assess the impact of a soccer match on the players involved.

Despite all this background, to our knowledge, no study has yet focused on the individual analysis of HRV in professional soccer players in a national team during training camps. Hence, the objectives of this study were (1) to introduce a method to analyze individual HRV responsiveness after playing a soccer match; (2) to determine whether the proposed method is feasible for classifying players as High-, Low- or Non-responders; and (3) to determine whether traditional group changes analysis produces similar results to a group analysis based on individual responses.

## Results

### Between groups HRV changes

**Figure 1:**
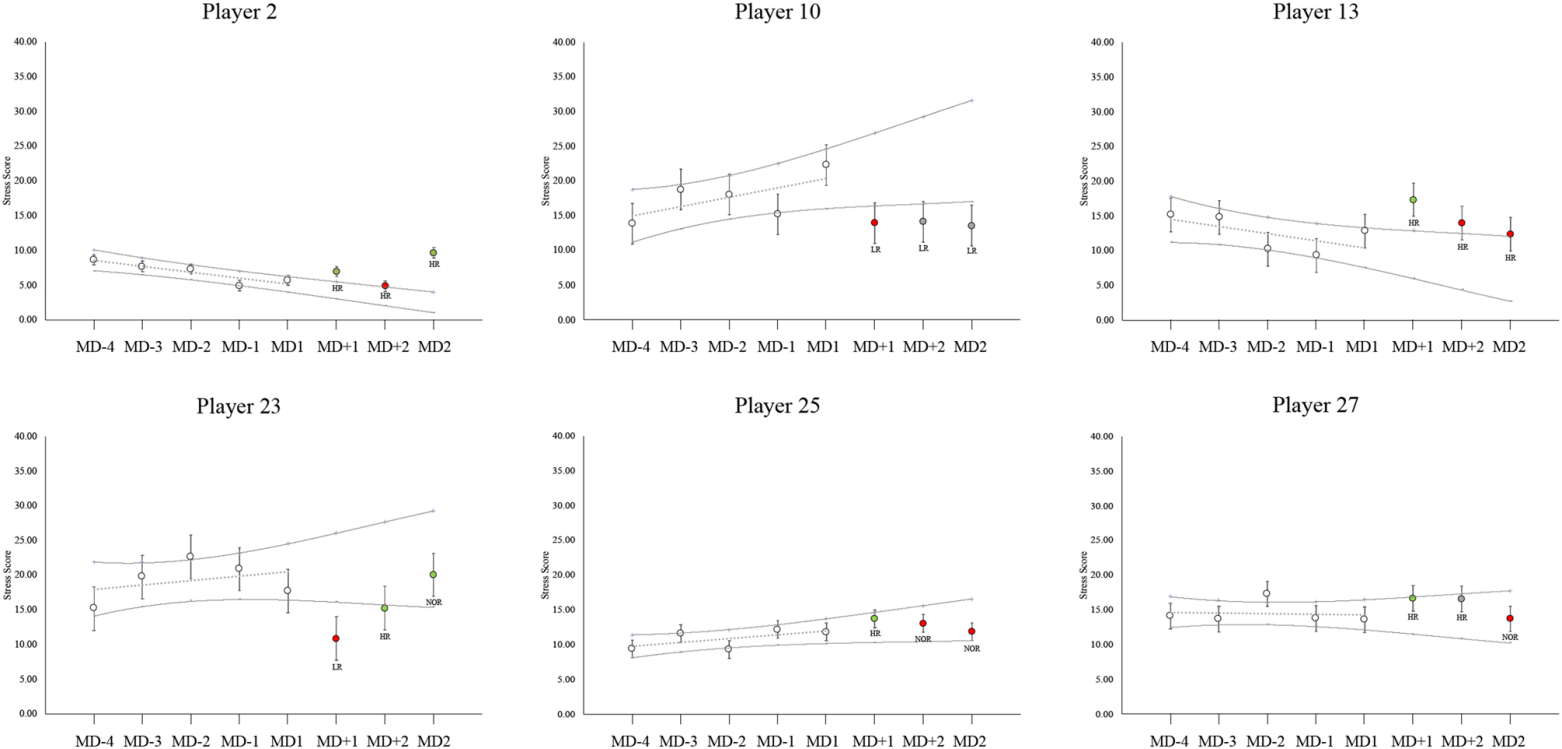
Random individual players’ showcases for LnRMSSD time course over a single training camp. Grey dotted line shows individual baseline trends. Horizontal lines represent the individual trivial band zone for each player, calculated as the individual Smallest Worthwhile Change. Vertical lines for each data time point represents the individual Typical Error of Estimate. White points = baseline values. Green points: high-responder from the previous day. Red points: low responder from the previous day. Grey point: non-responder from the previous day. *HR* high-responder from baseline trend. *LR* low-responder from baseline trend. *NR* non-responder from the previous day.

**Figure 2:**
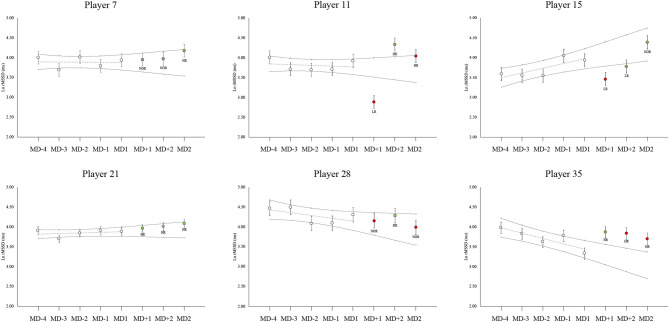
Random individual players’ showcases for Stress Score time course over a single training camp. Grey dotted line shows individual baseline trends. Horizontal lines represent the individual trivial band zone for each player, calculated as the individual Smallest Worthwhile Change. Vertical lines for each data time point represents the individual Typical Error of Estimate. White points = baseline values. Green points: high-responder from the previous day. Red points: low responder from the previous day. Grey point: non-responder from the previous day. *HR* high-responder from baseline trend. *LR* low-responder from baseline trend. *NR* non-responder from the previous day.

**Figure 3:**
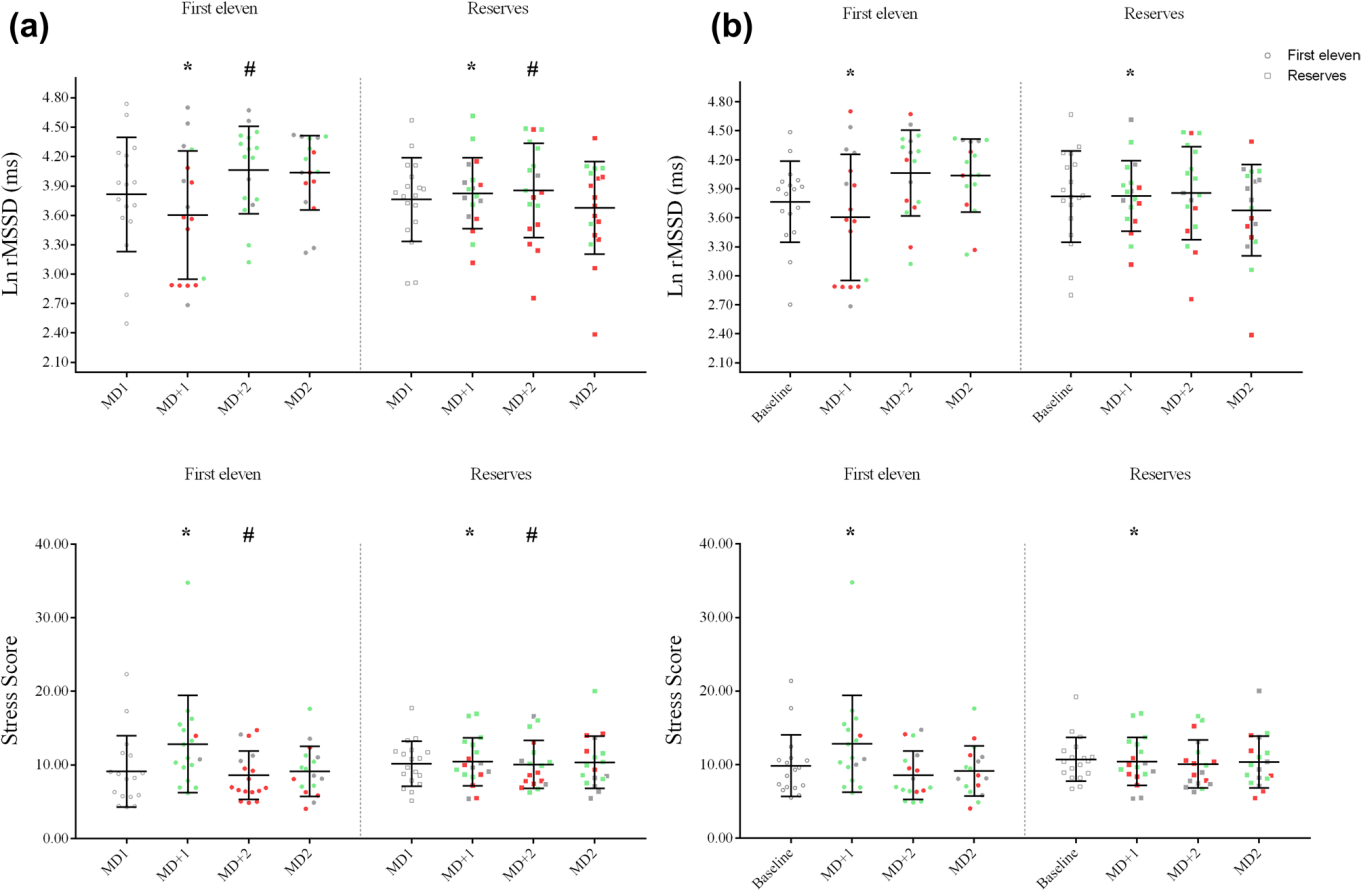
Descriptive group data and individual responsiveness for First Eleven and Reserves groups using the day-to-day method (**a**) and baseline method (**b**) of analysis for LnRMSSD and Stress Score. Horizontal lines show group mean plus standard deviations. Grey colored circles = Non-Responders. Green colored circles = High-Responders. Red colored circles = Low-Responders. * = significant changes at *p* < 0.001. # = significant changes at *p* < 0.05.

Tables 1 and 2 show individual and group HRV changes for both methods of analysis used in the First Eleven and Reserve groups, respectively. LnRMSSD smallest worthwhile change (SWC) values for First Eleven and Reserves during baseline were 2.42 ± 1.12% and 2.53 ± 0.98, respectively. With regard to SS, the SWC for First Eleven and Reserves at baseline was 9.44 ± 3.99% and 7.17 ± 3.32%, respectively. Visual differences were observed in the individual trivial band for each player, in addition to different individual baseline trend direction in both groups for LnRMSSD (Fig. 1) and SS (Fig. 2). Both methods showed substantial individual changes, regardless of the group. Figure 3 shows individual and group HRV values, together with within-group changes for First Eleven and Reserves. There was a significant Time*Group interaction from match-day (MD)1 to MD + 1 in LnRMSSD (F_1,33_ = 4.91, *p* = 0.034, η_2_p = 0.13–moderate) and in SS (F_1,33_ = 5.91, *p* = 0.021, η_2_p = 0.15–large) using the day-to-day method. Next day (MD + 1 to MD + 2) also showed a significant Time*Group interaction in LnRMSSD (F_1,33_ = 8.79, *p* = 0.006, η_2_p = 0.21–large) and in SS (F_1,33_ = 8.09, *p* = 0.008, η_2_p = 0.20–large) using this method. The baseline method showed similar interactions in Time*Group from MD1 to MD + 1 in LnRMSSD (F_1,33_ = 5.99, *p* = 0.020, η_2_p = 0.15–large) and in SS (F_1,33_ = 7.79, *p* = 0.009, η_2_p = 0.19–large). The baseline method did not show significant interactions between MD + 1and MD + 2 in any of the HRV indexes.

**Table 1:** Individual and group changes from previous days and baseline for LnRMSSD and Stress Score in First Eleven group.

Player	Ln rMSSD						Stress score					
	Change from previous day			Change from baseline			Change from previous day			Change from baseline		
	MD + 1	MD + 2	MD2	MD + 1	MD + 2	MD2	MD + 1	MD + 2	MD2	MD + 1	MD + 2	MD2
1	=	?	=	?	?	=	↑	↓	=	↑	↑	↑
2	?	?	↓	=	=	↓	=	↓	↑	↑	↑	↑
3	↓	↑	=	↓	↓	↓	↑	↓	?	↑	=	↑
4	↓	↑	?	↓	↑	=	↑	↓	↑	↑	↑	↑
5	?	?	?	=	=	=	?	?	?	?	?	?
6	?	↑	?	?	↑	↑	↑	↓	↑	↑	?	↑
7	?	?	=	?	?	=	↑	?	↓	↑	↑	?
8	↓	↑	↑	↓	=	↑	↑	↓	?	↑	=	↓
9	=	↑	↓	↓	↑	=	↑	↓	↑	↑	↓	↓
10	↑	↑	=	↑	↑	↑	↓	?	?	↓	↓	↓
11	↓	↑	↓	↓	↑	↑	↑	↓	=	↑	=	?
12	=	↑	=	=	↑	↑	=	↓	=	=	↓	↓
13	↓	↑	?	↓	=	=	↑	=	=	↑	↑	↑
14	?	=	?	?	?	?	↑	=	↓	↑	↑	↑
15	↓	↑	↑	↓	↓	?	=	↓	↓	↑	=	=
16	?	?	?	?	?	?	↑	=	↑	?	=	?
17	↓	↑	=	↓	↑	=	↑	↓	?	↑	?	?
Group	↓ 0.026 (0.597)	↑ < 0.001 (− 0.978)	= 0.703 (0.094)	↓ 0.015 (0.686)	↑ 0.005 (− 0.784)	↑ 0.021 (− 0.620)	↑ 0.002 (− 0.732)	↓ < 0.001 (0.890)	= 0.285 (− 0.268)	↑ 0.006 (− 0.773)	↓ 0.023 (0.608)	= 0.253 (0.288)

↑) increase; ↓) decrease; =) non-substantial/significant change; ?= unclear change. Group changes are shown as *p* value (cohen-d).

**Table 2:** Individual and group changes from previous days and baseline for LnRMSSD and Stress Score in Reserves group.

Player	Ln rMSSD						Stress Score					
	Change from previous day			Change from baseline			Change from previous day			Change from baseline		
	MD + 1	MD + 2	MD2	MD + 1	MD + 2	MD2	MD + 1	MD + 2	MD2	MD + 1	MD + 2	MD2
18	↑	=	↓	↑	↑	?	↓	?	?	=	?	?
19	=	=	=	↑	↑	↑	↑	↑	?	↓	↓	=
20	↓	↑	↓	↓	=	=	↑	↓	↑	↑	↓	?
21	=	?	=	=	=	=	↓	↑	↓	↓	↓	↓
22	?	↑	↓	?	↑	?	=	=	=	?	?	↑
23	↑	↓	=	↑	↑	=	↓	↑	↑	↓	↓	?
24	=	↓	=	?	↓	↓	?	=	=	?	↑	↑
25	?	=	↑	=	?	↑	=	=	=	=	?	=
26	?	↑	↓	?	↑	?	↑	↓	?	↑	?	?
27	=	↓	↑	=	=	?	↑	?	↓	=	=	?
28	=	=	=	?	=	?	↑	↓	↑	↑	?	↑
29	?	=	=	=	=	?	=	↓	↑	=	↓	=
30	?	=	=	=	?	?	↓	?	=	=	=	↓
31	↓	↓	↓	↓	↓	↓	?	↑	↓	?	↑	?
32	=	↓	?	↓	↓	↓	=	↑	=	=	↑	↑
33	↓	↑	↓	↓	↓	↓	=	=	=	↑	↑	↑
34	?	=	↑	=	↑	↑	?	=	↓	?	=	↓
35	↑	=	=	↑	↑	↑	?	=	=	=	??	=
Group	= 0.488 (− 0.167)	= 0.744 (− 0.078)	= 0.073 (0.451)	= 0.495 (− 0.164)	= 0.319 (− 0.242)	= 0.734 (0.175)	= 0.697 (− 0.093)	= 0.638 (0.113)	= 0.682 (− 0.098)	= 0.710 (0.089)	= 0.267 (0.270)	= 0.488 (0.167)

↑) increase; ↓) decrease; =) non-substantial/significant change; ? = unclear change. Group changes are shown as *p* value (cohen-d).

### Within-group HRV changes

Parasympathetic vagal activity (LnRMSSD) changes were very similar at MD + 1 and MD + 2 using either of the analysis methods. There were significant changes in the First Eleven group using both analysis methods for these days (for MD + 1 the day-to-day method showed decreased LnRMSSD, *p* = 0.026, ES = − 0.597 moderate, and the baseline method showed decreased LnRMSSD, *p* = 0.015, ES = − 0.686 moderate; while for MD + 2 the day-to-day method showed increased LnRMSSD, *p* < 0.001, ES = 0.978 moderate, and the baseline method showed increased LnRMSSD, *p* = 0.005, ES = 0.784 moderate) (Table 1). No significant differences were found in the Reserves group (Table 2). However, at MD2, while the day-to-day method showed that two First Eleven players and three Reserves players had a substantial increase (First Eleven, Player 8 = 10.65% and Player 15 = 16.14%; Reserves, Player 25 = 7.77%, Player 27 = 19.81% and Player 34 = 8.52%), the baseline method showed a substantial increase in five First Eleven players (Player 6, Player 8, Player 10, Player 11 and Player 12) and in four Reserves players (Player 19, Player 25, Player 34 and Player 35). At MD2 there were only significant changes using the baseline method (increased LnRMSSD, *p* = 0.021, ES = 0.620 moderate) in the First Eleven group. No significant changes were observed in the Reserves group at MD2.

Similar to the parasympathetic vagal activity, the sympathetic vagal activity changes (SS) were very similar at MD + 1 using both methods of analysis. At this day, the First Eleven group had a significant increase in SS (Day-to-day method: *p* = 0.002, ES = 0.732 moderate, and baseline method: *p* = 0.006, ES = 0.773 moderate) (Table 1), while the Reserves group did not show any significant decrease (Table 2). However, at MD + 2, while the day-to-day method showed a substantial decrease in 15 players (11 players from the First Eleven and four players from the Reserves), only two players from the First Eleven (Player 9 and 12) had the same response using the baseline method, with a total of 8 players showing a similar result (three First Eleven players and five Reserves players). Despite this discrepancy using the individual methods, the First Eleven group had a significant decrease in SS at MD + 2 (Day-to-day method—*p* < 0.001, ES = − 0.890 moderate, and baseline method—*p* = 0.023, ES = − 0.608 moderate), while the Reserves group did not show any significant decrease. Discrepancies between the two methods were also found at MD2, despite non-significant changes in both the First Eleven and Reserves groups.

### Responsiveness classification

Table 3 shows players’ responsiveness distribution based on prior individual analysis. Using the day-to-day method, only for MD + 1 to MD + 2 was there a significantly different responsiveness distribution in the number of Low-responder players in LnRMSSD (First Eleven = 100% players, Reserves = 0% players). SS also showed a significantly different responsiveness distribution from MD + 1 to MD + 2, but in the number of High-responder players (First Eleven = 100% players, Reserves = 0% players). Using the baseline method, LnRMSSD showed a significantly different responsiveness distribution in the number of High-responder players from Baseline to MD + 1 (First Eleven = 90% players, Reserves = 10% players).

**Table 3:** Distribution of High-, Low- and Non-responders in changes from previous days and changes from baseline for LnRMSSD and Stress Score variables.

Variable	Responders	Changes from previous day									Changes from baseline								
		MD + 1 (Res-Fir. %/response %)			MD + 2 (Res-Fir. %/response %)			MD2 (Res-Fir. %/response %)			MD + 1 (Res-Fir. %/response %)			MD + 2 (Res-Fir. %/response %)			MD2% (Res-Fir. %/response %)		
		Res	Fir	*p* value	Res	Fir	*p* value	Res	Fir	*p* value	Res	Fir	*p* value	Res	Fir	*p* value	Res	Fir	*p* value
Ln rMSSD	High-responders	77.8/38.9	22.2/11.8	0.200	42.9/50.0	57.1/70.6	0.002	50.0/27.8	50.0/29.4	0.069	90.0*/50.0	10.0*/5.9	0.012	57.9/61.1	42.1/47.1	0.537	37.5/33.3	62.5/58.8	0.292
	Low-responders	35.7/27.8	64.3/52.9		100.0*/44.4	0*/0		66.7/66.7	33.3/35.3		31.3/27.8	68.8/64.7		50.0/27.8	50.0/29.4		55.6/27.8	44.4/23.5	
	Non-responders	50.0/33.3	50.0/35.3		16.7/5.6	83.3/29.8		14.3/5.6	85.7/35.3		44.4/22.2	55.6/29.4		33.3/11.1	66.7/23.5		70.0/38.9	30.0/17.6	
Stress Score	High-responders	37.5/50.0	62.5/88.2	0.077	100.0*/44.4	0*/0	0.003	53.3/44.4	46.7/41.2	0.526	39.1/50	60.9 82.4	0.117	35.7/27.8	64.3/52.9	0.331	50.0/38.9	50.0/41.2	1.000
	Low-responders	83.3/27.8	16.7/5.9		33.3/38.9	66.7/82.4		58.3/38.9	41.7/29.4		83.3/27.8	16.7/5.9		58.3/38.9	41.7/29.4		50.0/27.8	50.0 / 29.4	
	Non-responders	80.0/22.2	20.0/5.9		50.0/16.7	50.0/17.6		28.6/11.1	71.4/29.4		66.7/22.2	33.3/11.8		66.7/33.3	33.3/17.6		54.5/33.3	45.5/29.4	

*Res* Reserves group, *Fir* First Eleven group, *MD* Match Day. * = between groups significant classification.

## Discussion

The objective of this study was to analyze daily HRV individual responsiveness after playing a soccer match during soccer national team training camps, using two different methods. The results showed that there was a high individual variation in both day-to-day changes and changes from baseline, regardless of whether the players were in the First Eleven or Reserves group. When players’ responsiveness was classified, differences were observed in the number of Low-responders in the two groups using the day-to-day method and in the number of High-responders in the two groups using the baseline method. However, when group changes were analyzed, only the First Eleven showed changes using either the day-to-day method or the baseline method after playing a soccer match.

The value of conducting groups versus individual changes when monitoring physiological factors has been recently highlighted^27^. Previously, we have shown that during training camps for a national soccer team, LnRMSSD was not affected during the training days (baseline)^9^. In that study, a positive increase in LnRMSSD was observed only in the First Eleven players after playing a soccer match. Although this was an important finding, in the present study the results showed that important individual variations occurred after a soccer match, not only in the First Eleven players (Table 1), but also in the Reserves (Table 2). For example, both LnRMSSD and SS showed a significant, moderate to large Time*Group interaction using both analysis methods for MD + 1 (Fig. 3). Although only the First Eleven as a group showed a significant change (Table 1), responsiveness at MD + 1 was classified in relation to First Eleven and Reserves groups. There was a 38.9–27.8–33.3% distribution of High-, Low- and Non-responders respectively in the Reserves for this day, which suggests that 66.7% of the Reserves players had an individual substantial change at this day (Table 3). Moreover, the high SDs shown in both LnRMSSD and SS SWC suggest that individual responses were very variable (Figs. 1 and 2). In this regard, although seven First Eleven players had a substantial decrease in LnRMSSD at MD + 1, Player 10 (Fig. 1) from the First Eleven group had a substantial increase at MD + 1 (+ 18.40%), which is in agreement with the individual changes that three Reserves players experienced at this date. This individual change was in agreement with the range of positive substantial changes that 11 First Eleven players exhibited at MD + 2 (including Player 10, Fig. 1). This may have potential importance if this player is of interest to the technical staff during a match. In relation, the group analyses have been shown to be different from individual analyses in aerobic fitness^27^, suggesting that individual variations are likely related to adequate or suboptimal internal load. These results show that individual analysis is necessary in this kind of situation, because individual responses can be detected when group changes are contrary, or even not shown.

The analysis methods performed in this study included the SWC as threshold of change, which is a common practice when HRV is analyzed^11,13^. Typically, studies use a baseline (reference) to perform the day-to-day analysis method, either using a single day^13^ or a week of training^11,29^ to calculate the SWC. The SWC found in our study for LnRMSSD in both groups is in line with other data previously published^2^. In relation to SS, the SWC was much lower compared to the whole-season tracking of a professional soccer team (≈21.5%)^24^, but higher than that recorded by Proietti et al.^30^ in LnSS. The main differences in SS SWC compared to our study may be related to the calculation method used; while in our study we firstly calculated the individual SWC and then averaged these for each group, previous authors calculated directly from a data batch obtained from all the players together. In addition to the day-to-day method, we also used a baseline method to calculate changes from baseline. Our results showed that both methods are feasible for detecting individual changes after playing a soccer match, but with different individual responses, even for the same players, these being more notable when SS was analyzed. This could be related to the high variability shown in SS (SWC ≈ 10%) (Fig. 2). Some authors have pointed out the usefulness of daily HRV analysis because of the high day-to-day variability^2,17^, even in the LnRMSSD, which is considered the most reliable and stable HRV measure when time-domain is considered ^17,22^. Previously, it has been shown that day-to-day analysis (one-time point analysis) of HRV include a high random variation, making difficult its’ interpretation, although using weekly rolling averages can solve this issue^13,17^. In relation to that, the baseline method, which is proposed for training camps in this study, solves the daily HRV variation because it includes a total of 4 days for an HRV average, which is in line with prior recommendations^2,13,17^.

In line with our procedures, some other studies have also analyzed individual changes in HRV^3,11,13,25^. Options include the use of a trivial band, where changes are related to random noise and biological variation, rather than to physiological changes^2,10,17^. Rabbani et al.^3^ used the SWC to establish the trivial band and used a similar qualitative assessment based on Hopkins’ spreadsheets (Figs. 1 and 2). Using a day-to-day analysis method and calculating SWC from the days after playing a soccer match, they found that most of the individual responses were unclear, in contrast to our results. In rowing, Plews et al.^11^ established their trivial band using the SWC from the first week of light training. We previously showed, with similar data, that the training days during training camps for professional national team soccer players did not alter parasympathetic vagal activity in soccer players^9^, which is explained by a low training load (e.g. low to moderate intensities resulted in HRV being fully restored within 24 h)^31^. Hence, our training days can be considered similar to a week of “light” training. Using this approach and, in line with our results, differences in individual responses in the LnRMSSD were found even during the heavy training period prior to winning the 2015 Rowing World Championships^11^. The present study showed important individual variations in HRV responses, but not solely in the players who played the soccer match (Fig. 3).

To interpret athletes’ responsiveness, some authors have used categories described as High-, Low-^20^ and Non-responders^18^. Currently, different methods exist by which researchers examine differential responses to exercise^18^, but to our knowledge our study is the first to classify soccer players’ HRV responsiveness after playing a soccer match. A simple approach to determining responsiveness involves the use of the SD. Some authors use one standard deviation above or below the mean to indicate high and low responders, respectively^32^. In the present study, we chose the SWC and the typical error (TE) to determine players’ responsiveness, rather than the SD, because this measure includes both random noise errors and biological variations simultaneously. Previously, Buchheit et al.^33^ showed that when using the typical error vs the SWC calculated from Cohens’ principle (0.2) lead to the smallest and greatest standardized changes in related physiological aspects. In this study, they showed greater variations using the TE rather than SWC. However, we decided to combine both (SWC for trivial band and individual TE as error for each value) because it accounts for the variation in the measurement or test itself (TE) and the biological variation (SWC)^2^. This approach is different to a group analyses method, because a group analyses can use either TE or SWC to stablish a trivial band, but still have the group confidence limits to assess the magnitude of a change, considering the standard deviation. Therefore, one of the main problems in analyzing group responsiveness is that important individual responses can be missed, because there is a high variation in response around the mean changes^20^, even though around 32% of the normally distributed measurements fall more than one SD from the mean^34^. In our study, we observed this high variation in response. Our results also showed that, despite a significant decrease in First Eleven subjects from MD1 to MD + 1 (Table 1), the responsiveness classifications were not significantly different over this period between the First Eleven and Reserves groups (Fig. 3). The two methods used in this study classified the players’ responsiveness differently. Considering team changes (Table 1), the First Eleven group significantly differed in changes using the day-to-day method from MD1 to MD + 1 in both HRV indexes. However, the distribution of responders was not significantly different, despite a higher percentage of Low-responders being observed.

Team analysis is a traditional procedure and in this study, we used two methods to perform it: based on individual changes (for later classifying individual players’ responsiveness); and based on a typical group (team) analysis (using a *t *test). Comparing both procedures, classifying the individual responses only showed a difference between the First Eleven and Reserves groups at MD + 2 when the day-to-day method was used and at MD + 1 when the baseline method was used. The main reason for the differences found using both procedures and methods of analysis is the high variation in individual responses (Tables 1 and 2). Hence, using the individual responsiveness classification and then analyzing those responses as a group, provided important information at MD + 2, in relation to the significant Time*Group interaction shown in LnRMSSD and SS when using the day-to-day method, and to the significant Time*Group interaction shown in LnRMSSD when using the baseline method. The only difference between the procedures was at MD + 1, where the team analysis showed a significant Time*Group interaction, but no differences in individual responsiveness classification were found. This difference could be related to individual variation, as shown by the individual analysis, despite a higher percentage of High-responders in the Reserves group (+ 77.8%, Table 3) compared to the First Eleven group (= 22.2%, Table 3).

This study only covered a methodological issue in relation to HRV analysis, and more research is needed to understand the relationship between the observed changes and other important fatigue variables such as wellness measures^5,29^ and other physiological and mechanical outputs^14^. In addition, a larger sample is required to understand whether classifying responsiveness can substitute for traditional group changes based on t-tests, which is typical practice in many other studies.

## Conclusions

In conclusion, the day-to-day and baseline methods showed a high variation in individual responses, even if players were Reserves, after playing a soccer match. However, the baseline method showed individual changes in relation to First Eleven group changes. Both methods were able to differentiate between High-, Low- and Non-responders in a national soccer team after playing soccer matches during training camps, using the TE and SWC to establish a trivial band. This classification was observed even in Reserves players, despite the group analysis only showing significant changes after playing a soccer match in First Eleven players. This analysis can be performed using changes from day to day, or from a baseline composed of various previous training days. However, in agreement with previous authors, the baseline method may be more appropriate due to the observed daily heart rate fluctuations. In addition, using the baseline method, responsiveness classifications were distinguishable between First Eleven and Reserves players the day after playing a soccer match.

## Methods

### Participants

Thirty four male soccer players (25.3 ± 2.3 years; 179.3 ± 5.9 cm height; 74.5 ± 8.0 kg weight) from a European national soccer team participated in this investigation. All participants were involved in daily training routines and matches in their respective soccer teams and had sustained no injuries in the previous 6 months. Players were divided into a First Eleven group (players who started the match and played a minimum of 60 min) and a Reserves group (players who did not play or played less than 60 min). The Reserves were considered the control group. The First Eleven consisted of 17 participants (26.1 ± 2.8 years; 179.9 ± 7.9 cm height; 77.9 ± 3.2 kg weight, 82.3 ± 6.7 min played) while the Reserves group also consisted of 17 participants (24.9 ± 3.8 years; 177.8 ± 6.8 cm height; 73.4 ± 4.9 kg weight, 9.8 ± 10.16 min played). Written informed consent was obtained from each subject before participation. This study was approved by the Latvian Football Federation board involved and conducted according to the Declaration of Helsinki.

### Design and procedures

**Figure 4:**
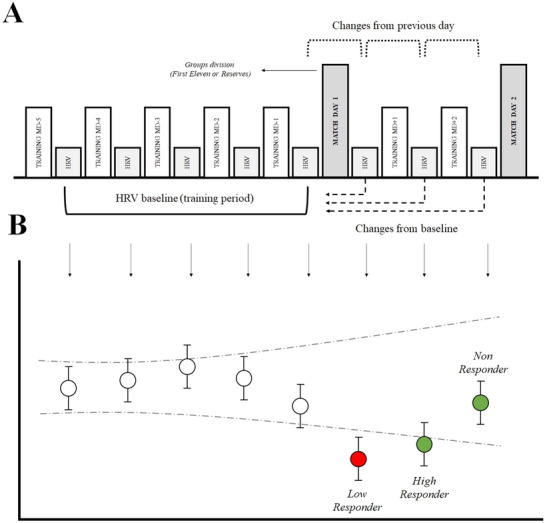
(**A**) Methodological design of each training camp intervention. Heart rate variability (HRV) measurements values from MD-4 to MD1 (baseline or training period) were used to calculate trivial bands for analyses. Dotted brackets represent changes from previous days. Dashed arrows represent changes from the baseline period. (**B**) Individual changes from previous days representation. Confidence bands show the limits of the trivial band, calculated as 0.5 times smallest worthwhile change. Vertical error bars represent the individual Typical Error of Estimate. White circles represent HRV values from baseline. When a change is substantially different from the previous day (day-to-day method), individual responsiveness is shown as low-responder (red) or high-responder (green). When a change is substantially different from the baseline (baseline method), individual responsiveness is shown un letters as low-, high-, or non-responder.

This investigation was an observational cross-sectional study. Players participated in two training camps in preparation for the France 2016 UEFA European Championship. The training camps consisted of a double match and were 9 days in duration (Fig. 4A). The training camp consisted of two main periods in relation to matches. The first period (training period) lasted for 6 days, while the second period (after match period) was 3 days. Matches took place on days 6 (MD1) and 9 (MD2). During the camp, players were subject to a strictly controlled routine. All the players had the same number of training sessions and intensity of training during the training period. Players were required to sleep a minimum of 7 h each night and to take a nap of between 20 and 40 min after lunch. Every morning, players were woken up for HRV measurements. Details of the trainings, training load and camp activities have been published elsewhere^9^.

### Heart rate variability measurements

Each morning, the medical staff woke the players individually and evaluated their readiness to train. None of the players suffered any illnesses or impediments to participating in the study. Approximately 20 min after waking up and emptying the bladder (between 8:00 and 9:30 am), the players were required to go to a silent, dimly lit room, at a comfortable temperature (between 20 and 24 °C), to perform the RR interval recording for a period of 10 min.

A Garmin HRM-Run heart rate monitor was used to record RR intervals (Garmin, Schaffhausen, Switzerland). The Garmin HRM-Run has been proved to be valid an reliable to measure HRV^9^. Data were extracted using Seego software (Realtrack Systems, Almeria, Spain) using an ANT + connection. Subsequently, raw data were exported and analysed using Kubios HRV software (University of Eastern Finland, Kuopio, Finland). For the analysis, only the last 8 min of the signal were considered. The first two minutes were used to stabilize the heart rate^13^. For further analysis, the LnRMSSD was used as marker of HRV. The SS index was used as a marker of sympathetic activity and it was calculated, according to Naranjo et al.^16^, as the inverse of longitudinal diameter (SD2) of the Poincaré Plot multiplied by 1,000.

### Statistical analysis

Data are shown as mean ± standard deviation. Individual daily HRV changes were analysed via magnitude-based inferences calculation^35^ using an Excel spreadsheet designed for this purpose^36^. Two different analysis methods were used to monitor individual changes after playing a soccer match, represented on Fig. 4.B: the day-to-day method was used to analyze the likelihood of a change between days from MD1; while the baseline method was used to analyse the likelihood of a change from the training period (baseline—from MD-4 to MD1) for each day. In both cases, the SWC and confidence limits of 90% of the TE were used to assess the magnitude of the change for each player, expressed as a percentage. The SWC was calculated during baseline as 0.5 times the standard deviation^2^ for each participant. The SWC was used to calculate the trivial band, in which a participant did not show changes beyond random error and biological variability (TE). For both methods, the quantitative likelihood that the change was greater or lower than the reference value was evaluated as: < 25% = “very unlikely”; 25–75% = “possible”; > 75% = “very likely”^36^. A change that could indicate both a possible substantial increase and a possible substantial decrease was marked as “unclear”^36^. A change higher than 90% probability was considered a substantial change. Players were classified as High-, Low- or Non-responders in the event of a substantial increase (high-responder), decrease (low-responder) or a non-substantial change (non-responder) for each method used.

In addition, based on individual responses, group HRV changes were analyzed using a 3 × 2 contingency table. The table was composed of horizontal lines to show the players’ individual responses (High-, Low- or non-responder) and vertical lines to show the player group (First Eleven or Reserve). Fisher’s exact test was used to compare this categorical data. In this analysis, data is shown as relative (as a percentage) and absolute (n) frequencies for each category. In addition, the Cohen’s d effect size (ES) was calculated, and the following qualitative scale was used: < 0.2 "trivial"; 0.20–0.59 = "small"; 0.6–1.19 = "moderate"; > 1.2 = "large”^36^.

For traditional group changes, a Shapiro–Wilk test of normality was used to test the data distribution. A two-way repeated mixed model ANOVA was used to analyze changes using both methods, using Time as a within-subjects factor and Group as a between-subjects factor (Time*Group). Effect size was calculated using the Partial eta-square score (η^2^_p_) and interpreted using the following scale: < 0.01 (trivial), 0.01–0.06 (small), 0.06–0.15 (medium) and > 0.15 (large). Between-group changes were also analyzed using a paired samples test to test changes from day to day or from baseline. Where there was a normal distribution of data, a Students’ *t* test for paired samples was used. In the event of a non-normal distribution, a Wilcoxon-w test for paired samples was used. The significance level was set at 0.05. Tests were performed using SPSS v. 22 for Windows (IBM SPSS Statistics, Chicago, IL).

## Data Availability

The data that support the findings of this study are openly available in OSF at https://osf.io/mk9da/, reference number mk9da and 10.17605/OSF.IO/MK9DA.
